# Reactive Oxygen Species and Targeted Therapy for Pancreatic Cancer

**DOI:** 10.1155/2016/1616781

**Published:** 2016-01-03

**Authors:** Lun Zhang, Jiahui Li, Liang Zong, Xin Chen, Ke Chen, Zhengdong Jiang, Ligang Nan, Xuqi Li, Wei Li, Tao Shan, Qingyong Ma, Zhenhua Ma

**Affiliations:** ^1^Department of Hepatobiliary Surgery, First Affiliated Hospital, Xi'an Jiaotong University, Xi'an 710061, China; ^2^Department of General Surgery, First Affiliated Hospital, Xi'an Jiaotong University, Xi'an 710061, China; ^3^Department of General Surgery, Second Affiliated Hospital, Xi'an Jiaotong University, Xi'an 710004, China

## Abstract

Pancreatic cancer is the fourth leading cause of cancer-related death in the United States. Reactive oxygen species (ROS) are generally increased in pancreatic cancer cells compared with normal cells. ROS plays a vital role in various cellular biological activities including proliferation, growth, apoptosis, and invasion. Besides, ROS participates in tumor microenvironment orchestration. The role of ROS is a doubled-edged sword in pancreatic cancer. The dual roles of ROS depend on the concentration. ROS facilitates carcinogenesis and cancer progression with mild-to-moderate elevated levels, while excessive ROS damages cancer cells dramatically and leads to cell death. Based on the recent knowledge, either promoting ROS generation to increase the concentration of ROS with extremely high levels or enhancing ROS scavenging ability to decrease ROS levels may benefit the treatment of pancreatic cancer. However, when faced with oxidative stress, the antioxidant programs of cancer cells have been activated to help cancer cells to survive in the adverse condition. Furthermore, ROS signaling and antioxidant programs play the vital roles in the progression of pancreatic cancer and in the response to cancer treatment. Eventually, it may be the novel target for various strategies and drugs to modulate ROS levels in pancreatic cancer therapy.

## 1. Introduction

Pancreatic cancer is the fourth leading cause of death in the United States. Significant malignant phenotype, delayed diagnosis, early metastasis, and poor outcome are characteristics of pancreatic cancer. The mechanisms of pancreatic cancer are still poorly understood. Recent studies focused on reactive oxygen species (ROS) reveal the roles of ROS in pancreatic cancer, which may achieve breakthrough in the therapeutic strategies.

ROS are substances with significant oxidative activity. Intracellular ROS oxidize lipids, proteins, and DNA, which leads to damage in various cellular organelles. It contains higher level of ROS in cancer cells of the nutrient-limited environment than in normal cells [1, 2]. It is consistent with our previous study that pancreatic cancer cells accumulated more ROS [3]. However, the ROS acts as a doubled-edged sword in pancreatic cancer. On the one hand, ROS-mediated DNA damage promotes the initiation of carcinogenesis and the malignant transformation of cells. At the same time, as signaling molecules, ROS can facilitate cell survival and cancer progression. On the other hand, excessive ROS promotes cytochrome c to be released into the cytoplasm and triggers programmed cell death [4]. The dual roles of ROS depend on its concentration. The regulation of redox homeostasis is necessary to maintain cellular function and ensure the survival of cells. While pancreatic cancer cells are evidenced by increased levels of ROS, the imbalance between ROS generation and elimination should be the only explanation.

Considering the dual roles, the strategies to decrease or increase ROS in cancer cells could be effective in cancer treatment. However, the preferred strategy should be based on the capacity of ROS elimination as well as the intracellular ROS levels. In fact, various drugs have been reported to control pancreatic cancer by targeting ROS. In this review, we will attempt to provide existing concepts on the role of ROS in pancreatic cancer and a deep understanding of targeting ROS as well as antioxidant programs for therapeutic strategies against pancreatic cancer and how this can be utilized in pancreatic cancer treatment.

## 2. Generation and Source of ROS

There are at least three types of ROS, including superoxide anion (O_2_^−^), hydrogen peroxide (H_2_O_2_), and the hydroxyl radical (OH^∙^) [5]. ROS can be generated by enzymatic reactions involving NADPH oxidases (NOXs), xanthine oxidase, uncoupled endothelial nitric oxide synthase (eNOS), arachidonic acid, and metabolic enzymes such as the cytochrome P450 enzymes, lipoxygenase, and cyclooxygenase [6]. For example, under physiological conditions, O_2_^−^ is generated by the one-electron reduction of O_2_ through NOXs in the cytosol. Besides, ROS can also be produced in the mitochondria by electron transport chain (ETC) complexes I, II, and III, in which the electron leaked from the respiratory chain may react with molecular oxygen [7–9]. When generated, the cytosolic O_2_^−^ is converted to H_2_O_2_ by the catalytic superoxide dismutase 1 (SOD1) within the cytoplasm and the mitochondrial intermembrane space. However, O_2_^−^ is converted to H_2_O_2_ by superoxide dismutase 2 (SOD2) in the mitochondrial matrix [10, 11]. In addition, H_2_O_2 _can also be produced as a byproduct of certain biochemical reactions, such as *β*-oxidation in peroxisomes and protein oxidation in the endoplasmic reticulum (ER) [10]. In the presence of metal cations, such as Fe^2+^ and Cu^+^, H_2_O_2_ can be further reduced into OH^∙^, an extremely reactive form of ROS with strong oxidizing potential, which is responsible for oxidative stress-induced cellular damage or genome instability [10].

## 3. Biological Functions of ROS

Low levels of intracellular ROS play an essential role in biological function. ROS is involved in cell cycle progression and cell proliferation [12]. Substantial studies have manifested that ROS can phosphorylate, directly react with, and modify structure of the signaling proteins, and thus they could participate in many signaling processes, including immune signaling, apoptosis, metabolism, aging, and hypoxic signaling [12, 13]. Notably, ROS can reinforce the epidermal growth factor (EGF) and platelet-derived growth factor (PDGF) mediated signaling pathways, in which EGF and PDGF activate the tyrosine kinase activity of their receptor tyrosine kinases (RTKs) and subsequently their autophosphorylation, resulting in activation of downstream signaling pathways, including phosphatidylinositol 3-kinase- (PI3K-) AKT signaling and the RAS-MEK-MAPK cascade [10, 14].

## 4. ROS and Pancreatic Cancer

ROS is tightly controlled by intracellular antioxidant programs. It has been known that cancer cells are characterized by an increased rate of ROS production, which enhances the tumor metabolic adaptation, proliferation, survival, and angiogenesis. Increased ROS production is also a hallmark of pancreatic cancer and ROS is suggested to be prosurvival and antiapoptotic in pancreatic cancer cells [15]. It has been reported that pancreatic cancer cells with lower levels of ROS are more resistant to chemotherapy [16].

Reverted activity of emergency pathway for ROS confrontation or clearance plays a sobering role in elevated ROS levels of cancer cells. In normal cells, several tumor suppressor genes, such as p53, FoxO, retinoblastoma (RB), p21, p16, and breast cancer susceptibility genes 1 and 2 (BRCA1 and BRCA2) [17], modulate cells faced with oxidative stress to adapt to the remodeled redox balance, thus preventing lipid peroxidation and oxidative damage to DNA and protein and resulting in hypomethylation through promoting the impression of antioxidative genes or enhanced transcription of proapoptotic genes. However, in the absence of wild type suppressor genes, especially p53, cancer cells shut down the emergency role of several antioxidative pathways in case of ROS accumulation [18]. Oncogene plays an important role in the increased ROS production by affecting different pathways. Mutant Ras results in an upregulated ROS level, contributing to the DNA damage and malignant transformation [19]. Oncogene expression (Raf, Myc, cyclin E, etc.) can silence tumor suppressor genes, which could further increase the ROS production [20]. Inflammation promotes the production of ROS in cancer cells. The release of superoxide anion, hydrogen peroxide, and hydroxyl radicals by macrophages and neutrophils is sustained by activation of plasma membrane NADPH oxidase [21]. In addition, there are also many other factors that can stimulate cancer cells to generate ROS, such as serum, insulin-like growth factor I, and fibroblast growth factor-2 [22].

Endogenous antioxidant compounds can be classified as enzymatic antioxidants and nonenzymatic antioxidants. Enzymatic antioxidants consisted of superoxide dismutase (SOD), catalase (CAT), glutathione peroxidase (GPx), and thioredoxin (TRX) family, while nonenzymatic antioxidants can be categorized into metabolic antioxidants and nutrient antioxidants [23]. Glutathione exists in two forms: reduced (GSH) and oxidized (GSSG) states. GSH, the most abundant antioxidant in the cell, participates directly in the neutralization of ROS. In this process, the thiol group of cysteine donates a reducing equivalent (H^+^ + e^−^) to ROS. Meanwhile, glutathione itself becomes reactive and readily reacts with another reactive glutathione to form GSSG. GSH can get reduced from GSSG by the glutathione reductase and NADPH [24]. Thus, an increased GSSG-to-GSH ratio is considered indicative of oxidative stress [25]. With increased ROS production, cancer cells can avoid the cell death and damage by upregulation of antioxidant programs.

*Threshold Concept of ROS*. Both normal and neoplastic transformed cells have the balance of intracellular ROS with antioxidant programs, which is essential for maintaining cell functions. However, any imbalance can result in a wide range of pathological changes. Generally, excessive ROS are harmful to cells, tissues, and organisms. When the concentration of ROS in the tumor microenvironment elevated and the capacity of the antioxidants adapted, it means that low-to-moderate levels of ROS production may enhance the proliferation of cancer cells by acting as signaling molecules or promoting the mutation of genomic DNA, which could result in oncogene activation as well as modifying gene expression and initiating neoplastic transformation [26, 27]. However, when the ROS levels exceed the optimum concentration, it can exhaust the available antioxidant programs. As a result, ROS can evoke irreversible oxidative damage and induce cell death via apoptosis, necrosis, and autophagy.

Thus, ROS has a dual role in carcinogenesis. Hypoxia is a characteristic feature of pancreatic cancer. It has been reported that HIF-1*α*, an angiogenic gene, can be promoted by ROS through the PI3K/Akt/p706K pathway [28]. Pancreatic cancer is characterized by a predominant desmoplastic response and the activation of PSCs (pancreatic stellate cells) is mainly responsible for the desmoplasia [29, 30]. Our previous study showed that ROS is an important mediator in the activation of pancreatic stellate cells (PSCs). With hypoxia, ROS may activate PSCs by stabilization of HIF-1*α* and upregulation of Gli1 expression, thus promoting PSCs to secret soluble factors such as IL-6, SDF-1, and VEGF-A to favor pancreatic cancer invasion [31]. Fiorini et al. [32] found that ROS acts as an adaptive strategy to inhibit “autophagic cell death,” and its antiautophagic effect may be mediated by upregulating AKT/mTOR pathway in pancreatic cancer. However, upregulation of intracellular ROS can promote chemosensitivity of cancer cells towards mTOR inhibitors, which is used in clinical trials currently [32]. Intracellular ROS can make pancreatic cancer vulnerable to extracellular turbulence such as chemotherapy and radiotherapy. By upregulating death receptor 5 expression, ROS can enhance cellular apoptosis induced by dihydroartemisinin, a semisynthetic derivative of artemisinin [33]. Previous study demonstrated that the JNK pathway in pancreatic cancer stem cells (CSCs) is upregulated and the JNK pathway inhibition can increase the intracellular ROS induced by chemotherapeutic agents such 5-FU and gemcitabine (GEM), indicating an important role for ROS in increasing chemosensitivity [34]. To summarize, the strategies to decrease or increase ROS in cancer cells would be effective in pancreatic cancer treatment, but the vital point is how to confirm the threshold of ROS and the ratio of ROS to antioxidant in order to select the right way.

## 5. Strategies and Drugs Targeted to Decrease ROS in Pancreatic Cancer Treatment

ROS-mediated DNA damage promotes the initiation of carcinogenesis. Wang et al. [35] found that ROS can promote carcinogenic metals (trivalent arsenic [As(III)] and hexavalent chromium [Cr(VI)]) induced colorectal tumorigenesis through Wnt/*β*-catenin signaling pathway. In addition, ROS is involved in carcinogenesis in various types of cancers including skin and oral cancer [36, 37]. Since the process of ADM, PanIN to PDAC is a result of accumulation for mutated genes, ROS may make significant contributions. For pancreatic cancer, using IHC with an antibody specific for cleaved LC3, an important marker for monitoring autophagy, Yang et al. [38] found that there is a minimal cytoplasmic staining of cleaved LC3 in the normal pancreatic ductal epithelium and low-grade PanINs; however, the high-grade PanIN-3 and PDAC showed upregulated cleaved LC3 staining intensity. Combined with another review of Yang and Kimmelman [39], they suggest that, in the stage of tumor initiation, from PanIN-1 to PanIN-3 until PDAC, the ROS level and DNA damage are increased as well as autophagy. Autophagy would initially suppress tumor initiation by maintaining cellular homeostasis to counteract the ROS-mediated DNA damage.

In addition to promoting the initiation of pancreatic cancer, ROS plays a vital role in the progression of pancreatic cancer. According to the threshold concept, mild rather than high ROS concentration tends to cause DNA damage which may lead to chromosome instability or gene mutation. To achieve cancer promotion, intracellular ROS has to be modulated properly to be maintained at mild concentration which is higher than normal cells but cannot be too high to kill cancer cells. Generally, ROS concentration is regulated by both production and degradation. In pancreatic cancer, ROS production is triggered by oncogenes, especially mutated K-Ras existing in over 90% of pancreatic cancer patients [40, 41]. Constitutional activation of Ras increased ROS production through activating NADPH oxidase 4 (Nox4), inducing cancer progression [42]. As the main resource of intracellular ROS, Nox4 is also upregulated by various growth factors [43], such as EGF [44] and TGF-*β*1 [45]. Besides growth factors, extracellular components, such as fibronectin and laminin, are also reported to positively modulate Nox4 in a 5-lipoxygenase-dependent manner [43]. Elevated levels of intracellular ROS promote the progression of pancreatic cancer in the following ways: (1) supporting cell proliferation and survival [42, 43]; (2) promoting endothelial cell proliferation to enhance angiogenesis via increasing the paracrine of IL-8 [46]; (3) inducing invasion and metastasis through processing EMT [47–52] and increasing the expression of MMPs [44, 45, 51]. In addition, ROS mediates hypoxia-induced pancreatic stellate cells activation to strengthen the malignant phenotypes [31].

However, antioxidant mechanisms in pancreatic cancer cells are involved in limiting the ROS concentration. Generally, ROS are firstly reduced to H_2_O_2_ by all SODs, including manganese superoxide dismutase (Mn-SOD), iron superoxide dismutase (Fe-SOD), copper/zinc superoxide dismutase (Cu/Zn-SOD), and nickel superoxide dismutase (Ni-SOD). In particular, Cu/Zn-SOD is an important player. Then, H_2_O_2_ is transformed to H_2_O by catalase and glutathione peroxidase. Studies have demonstrated that Mn-SOD expression is negatively related with cell growth because it decreases the level of intracellular ROS and inactivates the downstream signaling pathways. Interestingly, Mn-SOD was reported to exist only in the primary cancer cells but not in the metastatic cells [58], implying that this enzyme might prevent the progression of pancreatic cancer from obtaining more aggressive phenotypes. Another study showed that the overexpressed Mn-SOD leads to resistance of ROS-induced apoptosis and avoids host surveillance [59]. The mechanisms of Mn-SOD overexpression involve Mirk/Dyrk1B kinase [60] and Nrf2 transcriptional factor [61] which reduces ROS via upregulating antioxidant genes. Nrf2 is a transcriptional factor that can sense and respond to oxidative stress [62]. When integrating oxidative stress signals, the interaction between Nrf2 and Keap1 is disrupted, and Nrf2 located in the cytoplasm is stabilized and transported into the nucleus. Then, Nrf2 binds to antioxidant response elements (AREs) or electrophile response elements (EpREs) to cope with the stressed signals. Thus, antioxidant programs inhibit excessive ROS production to maintain pancreatic cancer cells at quiescent state, resulting in chemotherapeutic and radiotherapeutic resistance [63, 64]. Moreover, polymorphonuclear leukocytes- (PMN-) derived ROS also promotes tumor progression. Recurrence after surgery is an unsolved problem in pancreatic cancer therapy. Inflammation induced by surgery leads to PMN infiltration where higher level of extracellular ROS increases the expression of adhesive molecules in mesenteric cells [65] and endothelial cells [66, 67], facilitating local invasion and distant metastasis which accounted for cancer recurrence.

As previously described, mild ROS concentration promotes pancreatic cancer progression; therefore various studies focus on attenuating cell aggression via lowering elevated ROS. According to the mechanisms of ROS-induced cancer advancement, strategies are divided into four aspects: directly scavenging ROS by antioxidant agents, suppressing Nox4 or isoenzymes, blocking ROS-related signaling pathways, and inactivating stromal cells. Several drugs (Table 1) have been reported according to these strategies.

## 6. Strategies and Drugs Targeted to Increase ROS in Pancreatic Cancer Treatment

ROS are known as mediators of intracellular signaling cascades. These elevated ROS levels could increase the susceptibility of pancreatic cancer cells to cell death. Some activated signaling pathways, which increase intracellular ROS levels in cancer cells, make pancreatic cancer cells more vulnerable than normal cells to oxidative stress-induced cell death. This phenomenon is associated with some complicated mechanisms.

The PI3K/AKT signaling pathway is thought to play a prominent role in the proliferation of human cancer. The study conducted by Cheng et al. [55] showed that Longikaurin E (LE) induces apoptosis and antiproliferation properties in human pancreatic cancer cells and modulates the PI3K/AKT pathway. However, NAC, a kind of antioxidant, reversed all of these LE-mediated pharmacological activities. Pathania et al. [68] also reported that compound 3b exerts Akt-dependent ROS-mediated cell death. There are two major apoptotic pathways: intrinsic mitochondrial and extrinsic death receptor. Both the pathways contribute to the drug-induced death of cancer cells [69]. Caspases are a family of cysteine proteases and they have a pivotal role in the induction of apoptosis through cleavage of different substrates [70]. It has been reported that LE induces the apoptosis of pancreatic cancer cells through decreasing the Bcl-2/Bax and Bcl-xl/Bax ratios and activating caspase-3 mediated by increased ROS [55]. It also has been shown that spiclomazine induces the apoptosis of pancreatic cancer cells through activating caspase-3/caspase-9 by increased ROS [71]. A study showed that 4-tert-butyl-2-[(cyclohexylamino) methyl]-6-methylphenol (TBMMP, NSC number: 48160) increases the release of cytochrome c and the expression of Bax and activates caspase-3/caspase-9 through promoting the production of ROS [72]. In addition, there are also some other signaling pathways related with ROS-induced apoptosis in pancreatic cancer cells. Mackenzie et al. [73] reported that valproic acid derivative (P-V; MDC-1112) increases the level of mitochondrial ROS which triggers the apoptosis. Dando et al. [74] showed that genipin or UCP2 inhibition triggers ROS-dependent nuclear translocation of the glycolytic enzyme glyceraldehyde 3-phosphate dehydrogenase (GAPDH) and formation of autophagosomes. Besides, there are some drugs and treatments inhibiting proliferative ability of pancreatic cancer cells via ROS-mediated pathways.

It means that, for established pancreatic cancers, we can elevate the ROS levels to kill cancer cells, which is the main mechanism of many kinds of chemotherapies. However, Ju et al. [75] found that gemcitabine can induce the accumulation of ROS and meanwhile increase the capacity of antioxidant programs, which can in turn reduce the level of ROS as intrinsic resistance to treatment. This indicates that it is more efficient in cancer therapy to kill cancer cells with the decreased antioxidant capacity combined with elevated ROS level. Several drugs (Table 2) have been showed according to these strategies.

## 7. Conclusion

Cancer is a multistage disease containing initiation, promotion, and progression during its development. At the initial stage of pancreatic cancer, the increased ROS cause DNA damage which may lead to chromosome instability or gene mutation, resulting in the progression of cancer. Autophagy would suppress this progression by maintaining cellular homeostasis to counteract the ROS-mediated DNA damage. Thus, strategies and drugs targeted to decrease ROS would be effective in preventing the initiation of pancreatic cancer. In the promotion of pancreatic cancer, when the increased ROS concentration is below a specific threshold, the mild concentration of ROS tends to promote the progression of cancer. Meanwhile, malignant cells induce antioxidant programs to set a new redox balance, resulting in cellular adaptation. On the other hand, inflammation induced by surgery leads to PMN infiltration where higher level of extracellular ROS facilitates local invasion and distant metastasis which accounted for cancer recurrence. To summarize, pancreatic cancer with low ROS concentration and strong antioxidant programs, especially after surgery, should be treated with strategies and drugs targeted to decrease ROS.

Since extremely high levels of ROS can induce cell death, treatment of chemotherapy, radiation, and small molecule compounds all can increase the level of intracellular ROS to induce cancer cell death. The increased intracellular ROS levels could make pancreatic cancer cells more vulnerable than normal cells to oxidative stress-induced cell death. However, cancer cells have the ability to develop resistance to therapeutics by adapting to oxidative stress through inducing the antioxidant programs, such as activation and stabilization of Nrf2. We speculate that when the increased level of ROS reaches the certain limit, the programmed cell death of cancer cells would be triggered in some communities. However, in some other communities, such as cancer cells with strongly increased antioxidant programs, they would continue to survive. Therefore, molecules that mediate oxidative stress adaptation could be the important targets for anticancer therapies. On the other hand, pancreatic cancer cells with high ROS concentration when treated with strategies and drugs targeted to further increase ROS levels together with the inhibition of antioxidant programs would be more efficient in inducing cell death and the combination of promotion of ROS production and inhibition of antioxidant capacity may be the novel target for pancreatic cancer therapy.

## Figures and Tables

**Table 1:** Drugs targeted to decrease ROS.

Drugs	Targeted strategy	Mechanism	Reference
N-Acetyl-L-cysteine	Antioxidant agent	Scavenging ROS to inhibit proliferation and invasion	[53]

Anti-Duox2 monoclonal antibody	Nox suppressor	Neutralizing Nox isoenzyme to decrease ROS production	[54]

Longikaurin E	ROS-related pathway blocker	Inhibiting p38-MAPK and PI3K/Akt pathway to induce apoptosis	[55]

Nexrutine	ROS-related pathway blocker	Inhibiting STAT3/LC3/ROS	[56]

ACEI	Stromal cell inactivator	Decreasing ROS in PSC to inhibit pancreatic fibrosis	[57]

*α*-Mangostin	Stromal cell inactivator	Inhibiting ROS-induced PSC activation to attenuate pancreatic cancer cell invasion	[31]

**Table 2:** Drugs targeted to increase ROS.

Drugs or treatment	Mechanisms	Reference
Gemcitabine	Increasing ROS activated MST1 translocated to mitochondria and formed a complex with the local protein Cyp-D induced death of pancreatic cancer cells	[76]

Eriocalyxin B	Increase the intracellular ROS levels and regulating the MAPK, NF-*κ*B pathways	[77]

Compound 3b	Increase ROS by AKT activation promoted activation of stress kinases (p38/JNK) resulting in pancreatic cancer cell death	[68]

Artemisinin	Induce apoptosis via the generation of ROS and triggering binding of CD95L to CD95 receptor	[78]

Genipin	UCP2 inhibition triggers ROS-dependent nuclear translocation of the glycolytic enzyme glyceraldehyde 3-phosphate dehydrogenase (GAPDH), formation of autophagosomes, and the expression of the autophagy marker LC3-II	[74]

P-V; MDC-1112	Reduce STAT3 levels in the mitochondria by preventing its translocation from the cytosol and enhanced the mitochondrial levels of ROS which triggered apoptosis	[73]

Noninvasive radiofrequency treatment	Impair the function of mitochondria in cancer cells and increased ROS production	[79]

Green 1	Increase ROS production in mitochondria	[80]

SKLB316	Decrease the mitochondrial membrane potential and induce the generation of ROS in cells	[81]

Gemcitabine	Enhance selectively the expression of CXCL8 through ROS generation and NF-*κ*B activation	[82]

Withaferin A combined with oxaliplatin	Enhance mitochondrial dysfunction, inactivation of the PI3K/AKT pathway, and accumulation of intracellular ROS	[69]

Spiclomazine	Reduce the mitochondria membrane potential, elevated ROS, and activated caspase-3/caspase-9	[71]

Cerium oxide nanoparticles	Sensitization of pancreatic cancer cells to radiation by ROS production	[83]

Oleanolic acid	Arrests the cell cycle and induces apoptosis, possibly via ROS-mediated mitochondrial and lysosomal pathway	[84]

CDDO-Me	Enhance the production of ROS and inhibited the telomerase activity loss of mitochondrial membrane potential and release of cytochrome c from mitochondria ROS-dependent downregulated p-Akt, p-mTOR, and NF-*κ*B (p65)	[85, 86]

Belinostat	Increase ROS-induced transforming growth factor-beta-activating kinase 1 (TAK1)/AMPK association to activate AMPK	[87]

TBMMP	Increase cytochrome c release, reduced mitochondrial membrane potential, activated caspase-3, caspase-9, elevated ROS, and increased expression of Bax	[88]

Isoalantolactone	Induce ROS-dependent apoptosis through intrinsic pathway	[89]

Gallic acid	Activated caspase-3, caspase-9, and reactive oxygen species	[90]

Dihydroartemisinin	DHA enhances Apo2L/TRAIL-mediated apoptosis in human pancreatic cancer cells through ROS-mediated upregulation of death receptor 5 (DR5)	[33]

BML-275	Induce ROS generation, DNA damage, and apoptosis via inhibition of the AMPK pathway and by inducing G2/M arrest via a pathway independent of AMPK	[91]

Nickel nanowires	Induce ROS-mediated apoptosis	[92]

Fenretinide	Induce apoptosis and autophagy and that sensitivity appears to be mediated by enhanced ROS	[93]

Sulforaphane	Induce autophagy depending on ROS	[94]

Brucein D	Activate redox-sensitive p38-MAPK pathway and inhibition of NF-*κ*B antiapoptotic activity mediated by enhanced ROS	[1]

Artesunate	Induce ROS-mediated apoptosis	[95]

Nitric oxide-donating aspirin	ROS → MAPKs → p21 (cip-1) → cyclin D1 → cell death	[96]

Benzyl isothiocyanate	Activate ERK, JNK, and P38 at leading to the induction of apoptosis mediated by enhanced ROS	[97]

Arsenic trioxide and parthenolide	Induce reactive oxygen species generation and apoptosis via the mitochondrial pathway	[98]

Triphala	Phosphorylation of p53 and ERK induces apoptosis mediated by enhanced ROS	[99]

Capsaicin	Induce apoptosis through ROS generation and mitochondrial death pathway	[100]

Resveratrol	Damage mitochondrial function that leads to increased ROS, apoptosis	[101]
